# Cancer research across Africa: a comparative bibliometric analysis

**DOI:** 10.1136/bmjgh-2022-009849

**Published:** 2022-11-10

**Authors:** Miriam Mutebi, Grant Lewison, Ajay Aggarwal, Olusegun Isaac Alatise, Christopher Booth, Miska Cira, Surbhi Grover, Ophira Ginsburg, Julie Gralow, Serine Gueye, Benda Kithaka, T Peter Kingham, Lofti Kochbati, Jennifer Moodley, Sulma Ibrahim Mohammed, Alex Mutombo, Ntokozo Ndlovu, Christian Ntizimira, Groesbeck Preer Parham, Fiona Walter, Jeannette Parkes, Delva Shamely, Nazik Hammad, Janet Seeley, Julie Torode, Richard Sullivan, Verna Vanderpuye

**Affiliations:** 1 Department of Surgery, Aga Khan University, Nairobi, Kenya; 2 King's College London, Institute of Cancer Policy, London, UK; 3 Health Services Research & Policy, London School of Hygiene & Tropical Medicine, London, UK; 4 Department of Surgery, Obafemi Awolowo University Teaching Hospital Complex, Ile-Ife, Osun State, Nigeria; 5 Departments of Oncology & Public Health Sciences, Queen's University, Kingston, Ontario, Canada; 6 National Cancer Institute Center for Global Health, Rockville, Maryland, USA; 7 Radiation Oncology, University of Pennsylvania Perelman School of Medicine, Philadelphia, Pennsylvania, USA; 8 Department of Radiation Oncology, University of Pennsylvania, Philadelphia, Pennsylvania, USA; 9 NYU Grossman School of Medicine, New York, New York, USA; 10 American Society of Clinical Oncology, Alexandria, Virginia, USA; 11 Service d'urologie de l'Hopital General Idrissa Pouye, Dakar, Senegal; 12 KILELE Health Association, Nairobi, Kenya; 13 Surgery, Memorial Sloan Kettering Cancer Center, New York, New York, USA; 14 Abderrahmen Mami Teaching Hospital, Ariana El Manar University, Tunis, Tunisia; 15 University of Cape Town, Rondebosch, South Africa; 16 Purdue University System, West Lafayette, Indiana, USA; 17 University of Kinshasa, Kinshasa, Congo; 18 Faculty of Medicine and Health Sciences, University of Zimbabwe, Harare, Zimbabwe; 19 African Centre for Research on End-of-Life, Kigali, Rwanda; 20 World Health Organization, Geneve, Switzerland; 21 Department of Obstetrics and Gynecology, UTH-Women and Newborn Hospital, University of Zambia, Lusaka, Zambia; 22 Wolfson Institute of Population Health, Queen Mary University of London, London, UK; 23 Department of Radiation Oncology, University of Cape Town, Rondebosch, Western Cape, South Africa; 24 Faculty of Health Sciences, University of Cape Town, Rondebosch, Western Cape, South Africa; 25 Department of Medical Oncology, Queen's University, Kingston, Ontario, Canada; 26 Department of Global Health & Development, London School of Hygiene and Tropical Medicine, London, UK; 27 Global Oncology Group, King's College London, London, UK; 28 Institute of Cancer Policy, King’s College London, London, UK; 29 National Center for Radiotherapy Oncology and Nuclear Medicine and Korle Bu Teaching Hospital, Korle-Bu, Ghana

**Keywords:** health services research, cancer

## Abstract

**Introduction:**

Research is a critical pillar in national cancer control planning. However, there is a dearth of evidence for countries to implement affordable strategies. The WHO and various Commissions have recommended developing stakeholder-based needs assessments based on objective data to generate evidence to inform national and regional prioritisation of cancer research needs and goals.

**Methodology:**

Bibliometric algorithms (macros) were developed and validated to assess cancer research outputs of all 54 African countries over a 12-year period (2009–2020). Subanalysis included collaboration patterns, site and domain-specific focus of research and understanding authorship dynamics by both position and sex. Detailed subanalysis was performed to understand multiple impact metrics and context relative outputs in comparison with the disease burden as well as the application of a funding thesaurus to determine funding resources.

**Results:**

African countries in total published 23 679 cancer research papers over the 12-year period (2009–2020) with the fractional African contribution totalling 16 201 papers and the remaining 7478 from authors from out with the continent. The total number of papers increased rapidly with time, with an annual growth rate of 15%. The 49 sub-Saharan African (SSA) countries together published just 5281 papers, of which South Africa’s contribution was 2206 (42% of the SSA total, 14% of all Africa) and Nigeria’s contribution was 997 (19% of the SSA total, 4% of all Africa). Cancer research accounted for 7.9% of all African biomedical research outputs (African research in infectious diseases was 5.1 times than that of cancer research). Research outputs that are proportionally low relative to their burden across Africa are paediatric, cervical, oesophageal and prostate cancer. African research mirrored that of Western countries in terms of its focus on discovery science and pharmaceutical research. The percentages of female researchers in Africa were comparable with those elsewhere, but only in North African and some Anglophone countries.

**Conclusions:**

There is an imbalance in relevant local research generation on the continent and cancer control efforts. The recommendations articulated in our five-point plan arising from these data are broadly focused on structural changes, for example, overt inclusion of research into national cancer control planning and financial, for example, for countries to spend 10% of a notional 1% gross domestic expenditure on research and development on cancer.

**WHAT IS ALREADY KNOWN ON THIS TOPIC**

- Cancer research that is nationally relevant is crucial for improving affordable, equitable outcomes. The state of African cancer research has been the subject of narrative discourse but contemporary objective metrics to benchmark country performances across the continent are not available.

**WHAT THIS STUDY ADDS**

- This study provides in depth cancer research performance metrics across Africa, with a particular focus on sub-Saharan Africa. A wide range of performance and output data, including on author sex, enables deep benchmarking between African countries and in comparison to other regions around the world.

**HOW THIS STUDY MIGHT AFFECT RESEARCH, PRACTICE OR POLICY**

- The African Cancer Research Intelligence provided by this study should inform both national cancer control strategies across Africa to support research and also international policy towards supporting the building of research capacity and capability.

## Background

**Figure 1:**
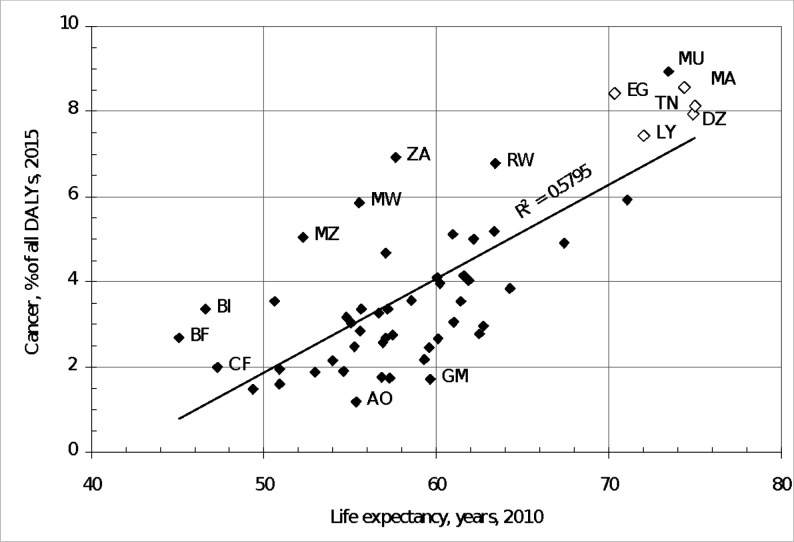
Disability adjusted life years compared to overall life expectancy. DALYs, disability-adjusted life years; AO, Angola; BI, Burundi; BF, Burkina Faso; CF, Centra African Republic; DZ, Algeria; EG, Egypt; GM, Gambia; LY, Libya; MA, Morocco; MU, Mauritius; MW, Malawi; MZ, Mozambique; RW, Rwand; TN, Tunisia; ZA, South Africa.

Africa is a large and diverse continent made up of 54 countries (online supplemental e-Table 1). While no pattern can adequately reflect the breadth and diversity of the African continent, there are estimated to be 198 distinct ethnolinguistic groups.^1^ There is a clear division in terms of human development index (HDI). The five North African countries (Algeria, Egypt, Libya, Morocco and Tunisia) are quite comparable in their demographic, economic and sociocultural background. They also share almost the same cancer risk and cancer protection factors, that is, level of industrialisation, control of infectious diseases, etc.^2^ Sub-Saharan African (SSA) countries, by comparison, are very diverse, spanning the HDI range from 0.72 for Botswana and 0.70 for Gabon and South Africa to just 0.35 in Niger.^3^ (The index is based on life expectancy, access to education and standard of living, see HDI—our World in data.) Of the 28 countries worldwide currently ranked by World Bank (2022–2023) as low-income economies, indicating they are the poorest countries in the world, 25 are located in Africa. Despite these development challenges, populations across Africa are rapidly ageing and non-communicable diseases, especially cancer (figure 1, online supplemental e-Table 2), are now a major challenge to health systems and economies.^4^

10.1136/bmjgh-2022-009849.supp1 Supplementary data

Context-relevant research is critical to driving more affordable, equitable and better outcomes through national cancer control planning across these diverse national systems in Africa.^5^ To date, policy research on African cancer control has been undertaken through a descriptive, narrative lens focused on care (outcomes)^6^ and qualitative work around research barriers.^7^ The literature has also examined new models for comprehensive cancer care^8^ and treatment^9^ but an objective assessment of the relative strengths and weaknesses of research at the country level has been absent. In the recent *Lancet Oncology* Commission on sub-Saharan Africa, authors noted the dearth of evidence-based polices and data to help build a new cancer research ecosystem across the continent, making the point that, ‘prioritisation of research needs and goals can be accomplished through stakeholder-based needs assessments as well as data-driven evidence’.^10^

To provide such a context-specific cancer research baseline for both national, regional and international cancer research systems strengthening, our comparative bibliometric multivariate analysis covers all countries in Africa but with a special emphasis on sub-Saharan Africa (the Middle East and North Africa region has already been the subject of a separate in-depth analysis).^11^ The aim was to examine national-level cancer research system trends, portfolio balance, focus, strengths and opportunities in order to provide underpinning strategic data for planning African cancer research strategies.

## Methods

### Deliberative coproduction and need assessment with stakeholders

The WHO Strategy on Health Policy and Systems Research entitled ‘Changing Mindsets’^12^ stressed that research should be demand driven and not viewed only as an activity. We undertook a deliberative demand-driven approach (arising from a recommendation by African authors working on the 2022 *Lancet Oncology* Commission Cancer in sub-Saharan Africa to develop better objective insights into the state of cancer research) to codevelopment and analysis as previously described by adapting virtual facilitation practices (group meetings via Zoom) using on-line decision workshops.^13^ The original concept and analytical design were codeveloped between authors led by a core African organisation from the African Organisation for Research and Training in Cancer (AORTIC) (MM, VV, JT, RS) through six iterative rounds with all coauthors. An open call to participate in this was launched in September 2020 with n=42 and n=31 pan-African participants in rounds 1 and 2. The current version of this manuscript was shared over a 4-month window (December 2021 to April 2022) to enable active feedback and all substantive participants and contributors were invited to be coauthors. A final meeting was undertaken in February 2021 to achieve consensus on the research recommendations.^14^

### Bibliometric analysis

We identified cancer research papers (articles and reviews) in the Web of Science (WoS) in February 2021 from 12 years, 2009–2020, by means of a complex search strategy or filter.^15^ This was based on several hundred specialist cancer journals and title words. These were each selected if 90% of the papers that they individually identified were marked as relevant to cancer research by a number of external experts whom we consulted over a period of over 25 years, although the filter was modified over time as additional specialist cancer journals were published and new title words appeared (mainly drugs and genes). The search included papers in any journal, including general medical and basic science journals, provided that they had a title term indicative of cancer (composed of 323 words and short phrases).^16^ This filter was developed through iterative rounds, which involved creating datasets and having these manually coded by clinical experts as to their relevance to the cancer research. This process gave a precision (or specificity), p, of 0.95 and a recall (or sensitivity), r, of 0.98, which are considered very high.

Papers were retained for analysis if they contained in their address 1 (or more) of the 54 African countries. (The WoS has included all the author addresses on papers since about 1973.) Their bibliographic details were downloaded file and converted into an MS Excel spreadsheet with a macro (programme) written by Philip Roe of Evaluametrics. A further macro identified the fractional count of each country for each paper. For example, a paper with two Egyptian and three Nigerian addresses would be categorised as EG=0.4, NG=0.6. For each African country, we analysed the numbers of published research papers for each year from 2009 to 2020 and calculated the average annual percentage growth rate (AAPG). We compared fractional country outputs during the last 5 years (2016–2020) with their wealth as measured by their gross national product in 2015 and multiplied by the percentage of their total disease burden in 2015 that was attributable to cancer. This timing provided an up-to-date view of African cancer research, and the interval between wealth measurement and research output allowed for the time needed to do research and publish the results.

In order to determine what percentage of each country’s biomedical research output was in the field of cancer, we then applied a second filter to the WoS. This was designed to identify such papers by means of words and contractions in their addresses, for example AstraZeneca, Bethesda, canc (cancer), dis (disease), Eisai (a pharmaceutical company), family, gene, hosp (hospital), INSERM, etc. We then compared the integer (or whole) counts of each country’s cancer research outputs in the 12-year period, 2009–2020, with its biomedical research output, and compared this ratio with the percentage of its total disease burden (measured in disability-adjusted life years (DALYs)) that was attributable to cancer in 2015. (This varied greatly and depended on the degree of development of the country, from 1.2% in Angola to nearly 9% in Mauritius.)

### Research domains, anatomical sites and levels

The African research publications were categorised into 12 research domains, such as genetics, paediatrics and surgery. These were defined with subfilters that each contained a set of title words and journal name strings to categorise relevant papers into particular domains. We also assigned the papers to some 16 manifestations of cancer (see table 1) by means of a further macro based on title words and some journal names (eg, *Lung Cancer, Breast Cancer Research*). Some papers were classified into more than one domain or anatomical site, but others could not be so classified. We then determined the research level of each paper (ie, whether paper is more basic or clinical), from clinical research (RL=1.0) to basic research (RL=4.0). This was based on the paper’s title words according to previously described methods.^17^ However, a minority of papers without a title word in either of the two lists (of clinical and basic words) could not be so classified.

**Table 1:** The sex determination of cancer researchers at country level (numbers of individual researchers), 2009–2020, for countries with at least 70 individuals

Country	M	F	U	F%	Country	M	F	U	F%
Mauritius	19	23	28	54.8	Rwanda	120	63	41	34.4
**Tunisia**	1833	2125	1157	53.7	**Libya**	115	60	68	34.3
**Algeria**	391	445	335	53.2	Uganda	351	172	97	32.9
Madagascar	42	38	67	47.5	Tanzania	245	119	156	32.7
South Africa	2066	1714	1336	45.3	Burkina Faso	121	56	81	31.6
Botswana	71	56	59	44.1	Togo	48	18	99	27.3
**Egypt**	10 243	7117	2459	41.0	Ghana	530	192	99	26.6
Africa	23 019	15 157	10 109	39.7	Cameroon	383	132	170	25.6
Mozambique	58	38	36	39.6	Sudan	334	107	112	24.3
**Morocco**	1947	1271	1163	39.5	Nigeria	1995	635	1125	24.1
Zimbabwe	87	56	83	39.2	Senegal	316	92	285	22.5
Zambia	74	47	76	38.8	Niger	42	8	20	16.0
Gabon	41	25	46	37.9	Mali	119	22	105	15.6
Malawi	164	98	108	37.4	Ethiopia	513	48	370	8.6
Kenya	551	298	185	35.1					

Country names in bold type are North African countries.

F%, per cent of sexed names that are female; F, females; M, males; U, unknown.

### The impact or influence of African countries’ cancer research

There are several ways in which the research outputs of a country’s scientists can be evaluated. One simple measure is the percentage of its papers that are classed as reviews, which are usually written by invitation from journal editors.^17^ A second is the relative importance of the journals in which the papers are published, measured as the average number of citations. However, because over half of these African papers are multinational, their journal impact factors (JIF) are likely to be increased over those of domestic papers, which give a better indication of a country’s capability. We have, therefore, calculated the mean JIF for both all papers and domestic ones.

The third measure is the number of citations. We calculated this for a 5-year window, beginning in the publication year, for papers from 2009 to 2016, from the annual citation counts for each paper that we downloaded from the WoS. This window was used as a compromise between the need for immediacy (ie, citations to recent papers) and stability (ie, inclusion of the peak year for citations, usually the second or third year after publication). This count was designated actual citation impact and was also determined for all of a country’s papers, and for its domestic ones. Finally, we determined how many of each country’s papers received enough 5-year citations (37 or more) to place it in the top 5% of African cancer papers for these 8 years. This percentage was normalised to 100 and designated as the worldscale value, by analogy with tanker shipping rates^pp^.

The 17 leading African countries were then ranked on each of these 6 indicators, and an overall ranking calculated as the sum of the individual ranks.

### The sources of financial support for the research

Since 2009, the WoS has included the full acknowledgement text as a searchable field, and has also listed the acknowledged funders of each paper in a separate column. We listed all of the funders in this column for five groups of countries and ranked them in descending order of numbers of acknowledgements:

- Five North African countries (Algeria, Egypt, Libya, Morocco, Tunisia).
- Twenty Francophone sub-Saharan countries.
- Five Lusophone (Portuguese-speaking) countries (Angola, Cabo Verde, Guinea-Bissau, Mozambique, São Tomé and Príncipe).
- Eighteen Anglophone countries.
- South Africa.

We needed to combine the many different name variants as they were not standardised.

Because so many of the papers were coauthored with countries in the Organization for Economic Coöperation and Development, much of the funding came from these countries. In particular, some acknowledgements were to the US National Cancer Institute and others to the National Institutes of Health without specification of which of some 27 individual institutes and centres had provided support.

### The author position and sex (or gender) of African cancer researchers

We used another macro to determine the country of affiliation of the first and last authors of all the papers with an address in sub-Saharan Africa. The intention was to see if African countries were prominent either in doing the majority of the work on each paper (first position) or the most senior (usually the last position). The identities of the first and last author on each paper were taken from the ‘authors’ column of the spreadsheet, not from the ‘addresses’ column. However, some author names were not listed in the address column.

We also wished to see if women were able to make a proportionate contribution to African cancer research, and how this varied by country. From the addresses’ column, which since 2009 in the WoS has tagged authors with their affiliations, we were able to generate lists of the names of authors from each African country. Most names included a given name although some just had initials. From previous studies, we had developed an extensive thesaurus of over 70 000 given names with their sex, provided that this was characteristic of over 75% of occurrences.^18^ However, many African given names were not listed, and we sought their sex from the commercial website, Gender-API, which can usually reveal the frequency of their occurrence in their database and the percentages of each sex. We were able to identify sex of 79% of the people by their names, including some with only initials if these matched those of names with the same surnames, country and given names without ambiguity. (For example, we assumed that Abanda, F. H. from Cameroon was male, because there was also a paper by Abanda, Fonbeyin Henry, who is male.)

## Results

### Cancer research outputs across Africa and in individual countries

**Figure 2:**
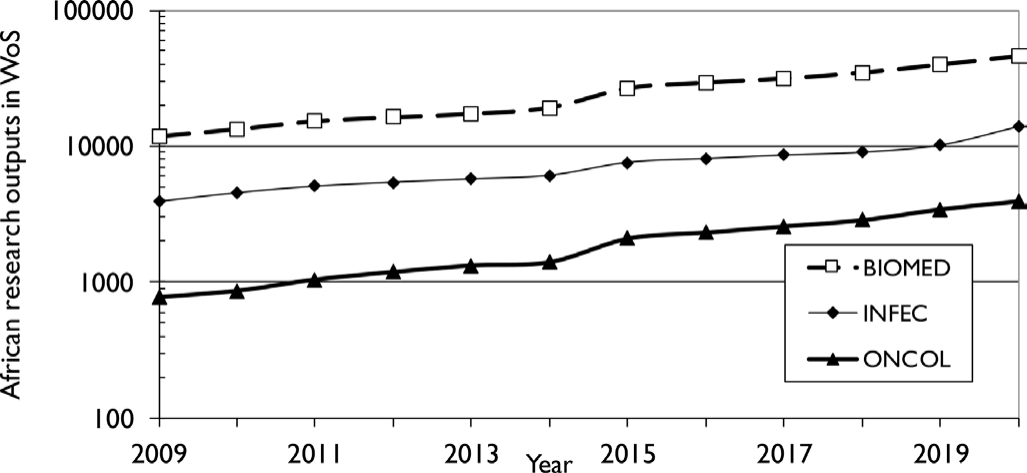
Research output domains in Africa. WoS, Web of Science.

We found 23 679 cancer research papers over the 12-year period (2009–2020) that contained an address from 1 or more African countries. On average, cancer research accounted for 7.9% of all African biomedical research outputs (figure 2); it increased from 6.5% in 2009–2010 to 8.6% in 2019–2020. For comparison, African research in infectious diseases is plotted on the same scale. In 2009–2011, its volume was 5.1 times than that of cancer research, but in 2018–2020 the ratio was only 3.2.

On a fractional count basis, the contribution from the African countries was 16 201 papers and the contribution from non-African ones was 7478 papers, or 31.6%. The 5 North African countries contributed 10 920 papers (67%) of the total on a fractional count basis. Of this total, Egypt published the most (n=7781; 48% of all African output). The 49 SSA countries together published just 5281 papers, of which South Africa’s contribution was 2206 (42% of the SSA total, 14% of all Africa) and Nigeria’s contribution was 997 (19% of the SSA total, 6% of all Africa).

**Figure 3:**
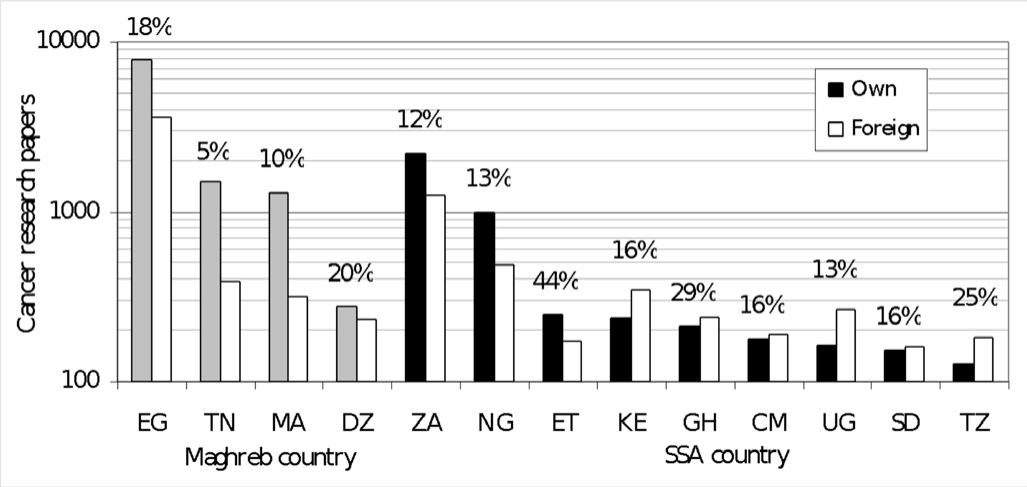
Distribution of country research by initiator. CM, Cameroon; DZ, Algeria; EG, Egypt; ET, Ethiopia; GH, Ghana; KE, Kenya; MA, Morocco; NG, Nigeria; SD, Sudan; SSA, sub-Saharan African; TN, Tunisia; TZ, Tanzania; UG, Uganda; ZA, South Africa.

The total number of papers increased rapidly with time, with an AAPG rate of 15%, much higher than that of the world cancer research output (7.8%) or that of the European Union (EU) (4.6%), but lower than that of China (19%). (There was a jump of almost 50% between 2014 and 2015 because of the increased coverage of the WoS of journals published in Africa and other non-traditional regions.) Most African countries have a dynamic and growing cancer research base with significant annual growth rates in their national outputs, for example, the top SSA countries achieved an overall average growth rate of 20% (range, 13% to 44%) (figure 3).

**Figure 4:**
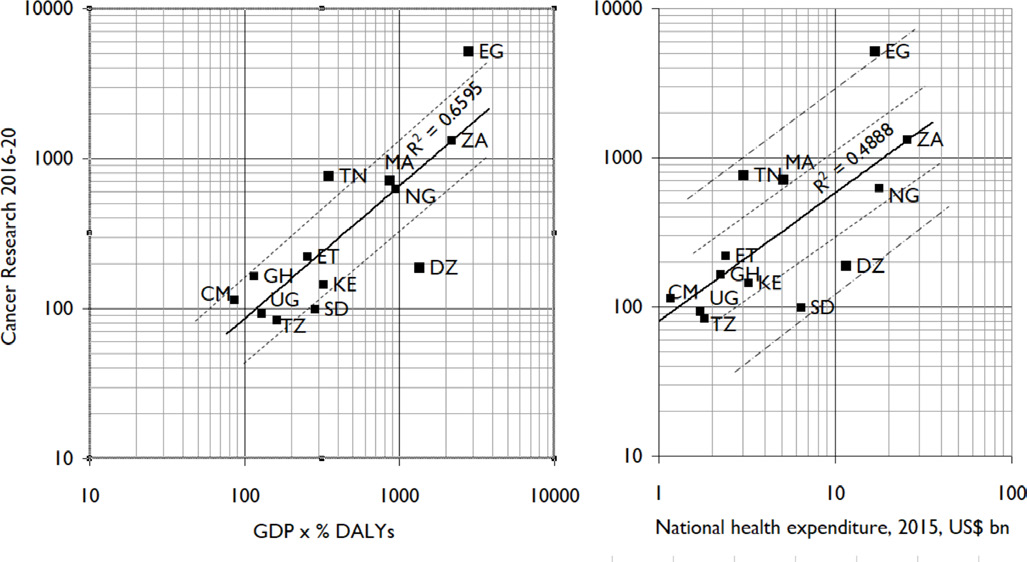
Research output versus country’s gross domestic product. CM, Cameroon; DALYs, disability-adjusted life years; DZ, Algeria; EG, Egypt; ET, Ethiopia; GDP, gross domestic product; GH, Ghana; KE, Kenya; MA, Morocco; NG, Nigeria; SD, Sudan; TN, Tunisia; TZ, Tanzania; UG, Uganda; ZA, South Africa.

**Figure 5:**
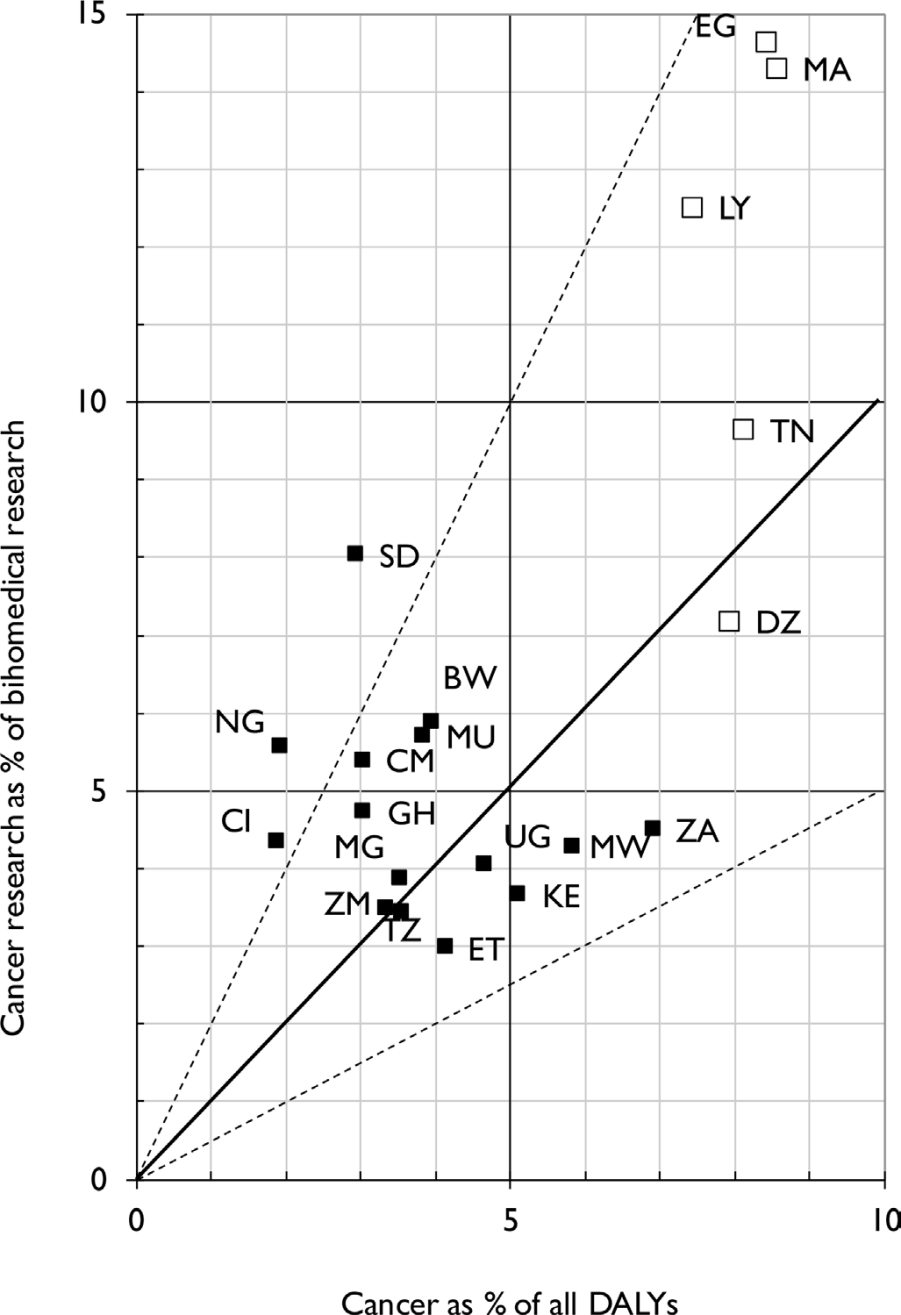
Comparison of research output versus disability adjusted life years. BW, Botswana; CM, Cameroon; DALYs, disability-adjusted life years; CI, Cote d'Iviore; DZ, Algeria; EG, Egypt; ET, Ethiopia; GH, Ghana; KE, Kenya; LY, Libya; MG, Madagascar; MU, Mauritius; MW, Malawi; NG, Nigeria; SD, Sudan; TN, Tunisia; TZ, Tanzania; UG, Uganda; ZA, South Africa; ZM, Zambia.

Country level outputs over this same period are shown in table 2. A comparison of the cancer research outputs of the highest output-producing countries relative to their wealth, measured by their gross domestic product (GDP), and multiplied by their overall disease burden as a percentage of all DALYs, is shown in figure 4. This figure also includes a plot of cancer research output relative to the countries' health expenditure. The correlation is fair but not as good as for European countries^19^ or Latin America.^20^ For example, North African countries, Egypt (EG) and Tunisia (TN), are clearly aligned in terms of the country’s GDP and their overall outputs. In SSA, both South Africa and Nigeria are similarly aligned, followed by a group of countries at the same development stage with major opportunities for expansion. The correlation between country-level cancer research outputs expressed as a percentage of DALYs is moderate (r^2^=0.70) (figure 5). However, there are significant differences between north and SSA countries, as well as between relative cancer research outputs for the same burden. For example, Egypt and Morocco (MA) published three times more cancer research relative to their biomedical research than Algeria (DZ).

**Table 2:** Cancer research outputs by individual African countries (2009–2020)

Country	INT	FRAC	% int'l	Country	INT	FRAC	% int'l
**Egypt**	**11 387**	**7781**	**31.7**	Mozambique	80	27.0	66.2
South Africa	3452	2206	36.1	Mauritius	61	25.4	58.4
**Tunisia**	**1894**	**1505**	**20.5**	Mali	66	25.1	62.0
**Morocco**	**1610**	**1293**	**19.7**	Congo	43	16.8	60.9
Nigeria	1486	997	32.9	Benin	39	13.1	66.4
**Algeria**	**506**	**274**	**45.9**	Guinea	19	10.2	46.2
Ethiopia	421	248	41.0	Angola	28	8.4	70.2
Kenya	578	233	59.6	Gambia	41	8.2	79.9
Ghana	452	213	52.9	Namibia	34	7.2	78.8
Cameroon	364	177	51.4	Central African Republic	15	6.4	57.6
Uganda	427	162	62.2	Eritrea	12	6.2	48.1
Sudan	314	153	51.3	Mauritania	8	5.5	31.2
Tanzania	306	125	59.2	Liberia	13	4.0	69.3
Senegal	200	114	42.9	Sierra Leone	12	3.6	70.3
Malawi	206	75.6	63.3	Burundi	11	3.4	69.4
**Libya**	**188**	**66.6**	**64.6**	Somalia	6	3.4	43.9
Côte D'Ivoire	113	62.0	45.2	Swaziland	8	2.0	74.5
Zimbabwe	130	54.4	58.1	Cape Verde	5	1.7	66.7
Zambia	121	48.4	60.0	Chad	5	1.3	73.4
Burkina Faso	103	46.7	54.6	Lesotho	2	1.0	49.4
Botswana	120	44.6	62.8	Djibouti	2	0.45	77.5
Rwanda	103	34.2	66.8	Comoros	2	0.34	82.9
Togo	54	33.8	37.3	Seychelles	5	0.28	94.5
Madagascar	70	32.7	53.3	Guinea-Bissau	1	0.14	85.7
Democratic Republic of the Congo	66	31.1	52.9	South Sudan	1	0.05	94.7

North African countries shown in bold.

FR, fractional counts; INT, integer counts; % int'l, percentage with international contribution.

### International collaboration in African cancer research

Both national and international collaborative cancer research is essential for the improvement of patient outcomes and the building of research capacity. For most countries, international collaboration, as measured by the presence of multiple countries among the addresses on the papers, was very high and for some, such as Uganda and Kenya, internationally coauthored papers were more than six times as numerous as purely national ones. Some international contributions were from other African countries, but mostly they came from Asia, Western Europe and North America. These varied between African countries (see online supplemental e-Figure 1). For example, two of the Francophone North African countries (Morocco, MA; Tunisia, TN) collaborated very little internationally, although they retained links with France, as did Algeria (DZ). Egypt (EG) collaborated mainly with Saudi Arabia, as did Sudan (SD). Nigeria’s preferred partner was Malaysia, reflecting significant diversity in international cancer research collaboration.

10.1136/bmjgh-2022-009849.supp2 Supplementary data

**Figure 6:**
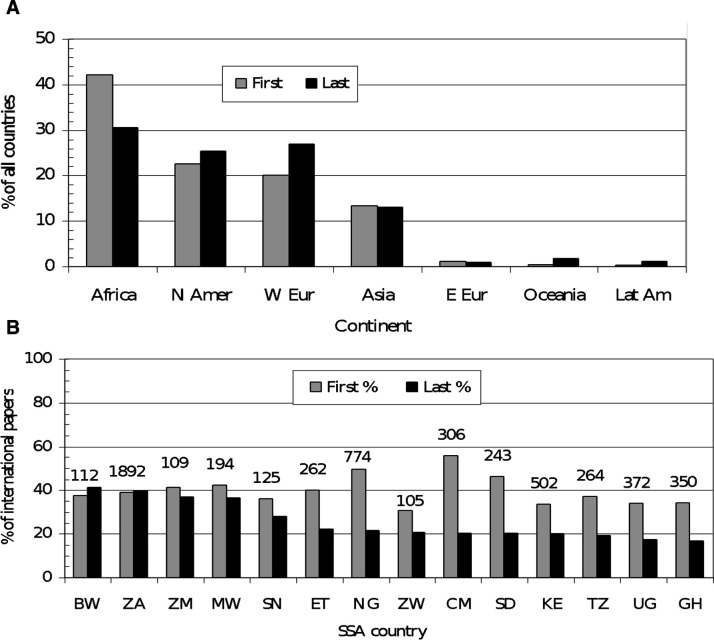
Distribution of authorship by position in different countries. BW, Botswana; CM, Cameroon; ET, Ethiopia; GH, Ghana; KE, Kenya; MW, Malawi; NG, Nigeria; SD, Sudan; SN, Senegal; SSA, sub-Saharan African; TZ, Tanzania; UG, Uganda; ZA, South Africa; ZM, Zambia; ZW, Zimbabwe.

To understand international collaboration in greater depth, we analysed the affiliations of the first and last authors of the international papers from the SSA countries (n=5303) (figure 6A). This figure shows that authors based in SSA countries were more likely to be first authors on these papers (42%) than last authors (30%). Of the last authors from SSA (n=1802) much the largest share (n=751, 42%) were based in South Africa. The last author position was dominated by authors from the USA (1347 papers, 25%). Western Europeans were also more likely to be last than first authors. The leading countries were the UK (413 papers, 7.8%), Germany (281, 5.3%) and France (248, 4.7%). However, there were very few coauthors from Central and Eastern Europe, Oceania or Latin America.

Authorship position in African countries is more heterogenous than Western convention based on first and last authors. However, an analysis of such positions does provide an understanding of the nature of international collaborations. Researchers from Botswana (BW) and South Africa (ZA) both held 40% of last author positions for their international papers, but this ratio is closer to 20% for most of the other SSA countries. Several of them had high levels of first author positions on their international papers, notably Cameroon (CM), with 56%, and Nigeria (NG), with 50%, but for most countries the levels were below 40% (figure 6B).

### Indicators of African cancer research influence or impact

Table 3 shows the different measures of influence or impact for the 17 leading African countries in terms of output. They are ranked by their positions on six different indicators, two of which are for their purely domestic papers (without international contributions) and four for all their cancer research papers. Although North African countries have high outputs, the impact of their cancer research papers is relatively low. This is especially true for the three Francophone countries, whose overall rank is lower than that of the other SSA countries listed.

**Table 3:** Different measures of impact and/or influence of the cancer research papers from 17 leading African countries

Country	ISO2	% revs	JIF dom	JIF all	ACI dom	ACI all	WS all	Rank
South Africa	ZA	15.8	2.3	3.7	8.7	10.5	80	23
Ethiopia	ET	14.5	2.4	3.4	9.4	10.2	95	25
Uganda	UG	10.6	2.1	5.7	6.4	11.9	75	28
Cameroon	CM	11.5	1.7	3.1	8.2	12.5	137	33
Zambia	ZM	13.7	1.8	5.6	0.0	11.4	70	39
Kenya	KE	12.6	1.7	4.8	6.3	10.3	67	40
Ghana	GH	14.8	2.2	4.4	4.9	8.4	51	40
Tanzania	TZ	9.7	1.7	3.5	6.3	11.0	101	41
**Egypt**	**EG**	**6.1**	**2.2**	**2.9**	**8.7**	**10.1**	**72**	**49**
Sudan	SD	12.9	1.5	2.8	4.7	7.2	61	61
Zimbabwe	ZW	9.1	1.4	5.9	3.4	6.1	66	62
Nigeria	NG	10.6	1.4	3.1	4.0	5.8	21	74
Botswana	BW	9.5	1.1	4.3	0.8	9.9	0	75
Namibia	NA	8.7		4.7	0.0	7.5	0	81
**Algeria**	**DZ**	**5.2**	**1.3**	**2.9**	**3.9**	**6.6**	**51**	**82**
**Tunisia**	**TN**	**7.3**	**1.5**	**2.3**	**4.2**	**5.6**	**28**	**82**
**Morocco**	**MA**	**12.8**	**1.3**	**2.3**	**2.2**	**3.1**	**8**	**83**

North Africa countries: bold type; sub-Saharan African countries: roman type.

ACI, mean 5 year citation count; dom, domestic papers without international collaboration; JIF, mean journal impact factor; overall rank, sum of individual rankings for six indicators; % revs, percentage of reviews; WS, worldscale value for top-cited 5% of papers (with ACI=37 or more).

The research level of the papers (a measure of how clinical or basic the research is) averaged 1.95, which varied very little over the 12-year study period (online supplemental e-Figure 2). However, it did vary greatly by country both for domestic papers and for international ones. For all the countries studied, international papers were more basic than domestic ones (though for South Africa, ZA, the difference is marginal).

10.1136/bmjgh-2022-009849.supp3 Supplementary data

### Research on different cancer anatomical sites and in different domains

**Figure 7:**
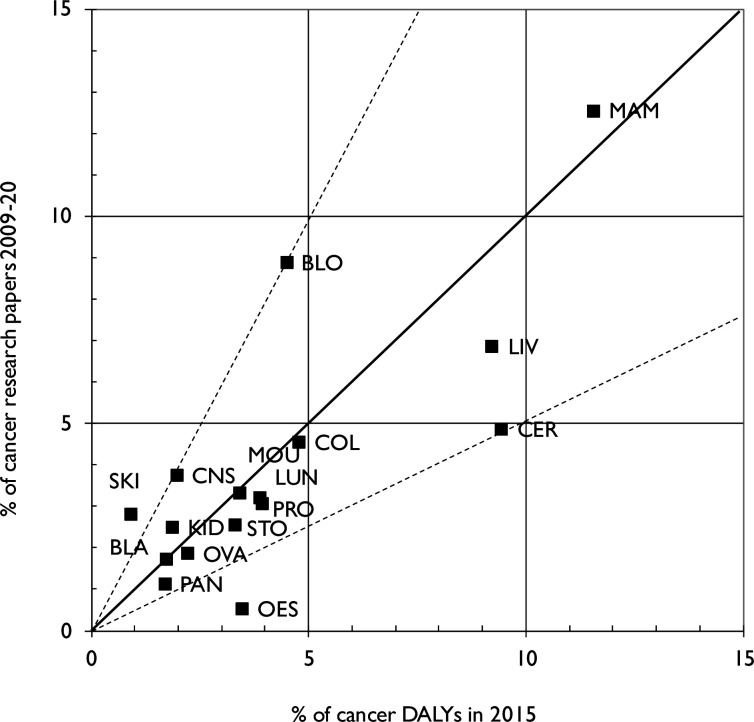
Percentages of SSA cancer research outputs on different cancers, 2009-20, versus the percentages of the total African cancer disease burden in 2015 (WHO data). *Diagonal line represents equivalence; dashed lines represent output either twice or half the amount corresponding to the burden*. BLA, bladder; BLO, Blood; CER, cervical; COL, bowel; CNS, brain; DALYs, disability-adjusted life years; KID, kidney; LIV, Liver; LUN, Lung; MAM, breast; MOU, head & Neck; OES, oesophageal; OVA, ovarian; PAN, pancreatic; PRO, prostate; SKI, malignant melanoma; STO, gastric.

The next analysis was of the distribution of the papers across the different anatomical sites. Figure 7 shows the output on each of the 16 listed in table 1 as a percentage of the total (23 679 papers) compared with the percentage of the overall cancer disease burden for all SSA. The correlation is moderate (r^2^=0.61). As in high-income settings, breast cancer (MAM) is the site-specific cancer with the highest research outputs. Blood cancers, including leukaemia (BLO), are heavily researched by a factor of two; again reflecting what is seen in high-income settings such as Europe.^19^ Research outputs that are proportionally low relative to their burden across Africa are cervical (CER), oesophageal (OES) and prostate (PRO) cancer.

**Figure 8:**
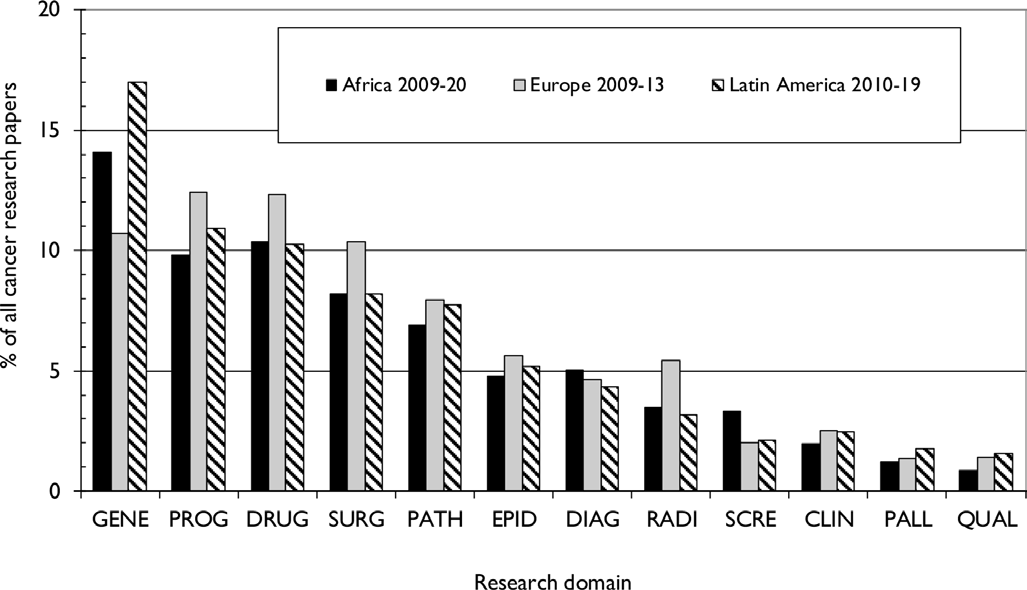
Research outputs by cancer domain. CLIN, clinical research; DIAG, early diagnosis; DRUG, systemic therapy; EPID, epidemiology; GENE, genetics; PALL, palliative care; PATH -laboratory medicine; PROG, biomarkers; QUAL, quality of life; RADI, radiotherapy; SCRE, screening; SURG, surgery.

Figure 8 shows the distribution of SSA research outputs by cancer research domain, compared with the distributions in Europe and in Latin America.^19^ These distributions are similar, although Africa did relatively more work on basic discovery cancer science and genetics (GENE) and screening (SCRE) than Europe, but less on the three major treatment modalities (systemic therapy (DRUG), surgery (SURG) and radiotherapy (RADI). Proportionally, very little research is carried out on palliative care (PALL) or quality of life (QUAL), a common finding in most countries around the world.

Paediatric cancer research in Africa accounted for 388 papers, or only 1.6% of the total. This compares with the much higher burden of cancer on African children, which in 2015 was 15% of total cancer DALYs in the five North African countries and 17% in SSA. (These ratios are much higher than those of 1.5% in North America and 1.0% in Western Europe.) African paediatric cancer research output more than doubled from 20 papers per year in 2009–2014 to 44.5 in 2015–2020, but as a percentage of the total it declined from 2.02% in 2009–2010 to 1.47% in 2019–2020. Its volume was clearly disproportionately small compared with the disease burden of cancer on African children. Such research as there was focused on eye cancers (retinoblastomas), with more than a quarter (n=97) of the papers, followed by leukaemia and cancers of the peripheral nervous system (55 papers each).

We further analysed the site-specific and research-domain focus by individual country (online supplemental e-Tables 6 and 7). This shows a complex mosaic of strengths and opportunities for expansion of research at country level. For example, while overall research outputs across all African countries on cervical cancer may be low, the relative commitments of some countries, for example, Nigeria, Ethiopia, Kenya, Ghana, Cameroon, Uganda and Tanzania are high (online supplemental e-Table 6). In contrast, the North African countries are uniformly weak in their research on cervical cancer.

### Sex (or gender) balance of African cancer research

There was a total of 82 118 individual contributions from researchers with an address in Africa, and we estimated that these came from 48 285 different individuals. Of these, we were able to determine that 23 019 were male names, 15 157 were female names and 10 109 were undetermined. They represented only 21% of the total, thanks to the Gender-API website and to correspondents in three countries (Egypt, Ghana and Nigeria) who were able to sex most of the relatively unusual names, a few of which were misprinted in the WoS because of the optical character recognition system that it used. Of the names that could be sexed, almost 40% were female. This compares with a world average of 36% in 2019, and indicates that female cancer researchers in Africa as a whole have made progress, although this average hides considerable country-level heterogeneity from 56% female cancer researchers in Mauritius (MU) to 10% in Ethiopia (ET) and 9% in Mali (ML) (table 1). The North African countries, notably Algeria (DZ), Tunisia (TN) and Egypt (EG), score well, but most of the other countries in the first column are Anglophone, and the majority in the second column are Francophone.

We also analysed the sex distribution of authors in first position (online supplemental e-Table 9), last position (online supplemental e-Table 10) and sole authorship papers (online supplemental e-Table 11). Despite the significant distributional differences between the best and worst performing countries, some such as South Africa (ZA) and Mozambique (MZ) consistently performed well in terms of their equitable gender balance in these significant author positions.

### The funding of African cancer research

North African countries enjoy significant overseas support from the USA (NCI/NIH), the EU and some major European national funders from countries such as Germany and the UK. There are, of course, national domestic funders and wider Middle East bodies (online supplemental e-Table 12A). In sub-Saharan Africa, the leading country, South Africa, is supported by not only domestic funding but also international funders, particularly ones in the USA, the EU and the UK (online supplemental e-Table 12D). The NCI/NIH, USA the EU and the UK’s Medical Research Council are also significant funders of cancer research across SSA. Anglophone SSA countries have the major share of this support, followed by Francophone countries (online supplemental e-Table 12B–D).

## Discussion

### Increasing intra-African cancer research

Despite its immense size, the cancer research output of countries across Africa contribute only 1.3% to global outputs compared with 2.9% of global GDP. This reflects long term underinvestment in science and technology, generally. African countries contribute only 1.1% to world total health research and development expenditure.^21^ Furthermore, in 2006 in Khartoum, African Heads of State and Government committed to raising their national gross domestic expenditure on research and development (GERD) to at least 1% of GDP.^22^ Yet, most African countries have failed to achieve this even before the impact of COVID-19. While there has been a welcome increase in cancer research over the last decade, this is unevenly distributed. Just 13 of 54 African countries were responsible for around 90% of total cancer research, with two countries contributing two-thirds of the entire continent’s cancer (and NCD) research, namely Egypt and South Africa.^23 24^ This critical finding speaks to the need for each country to develop a strategic plan to build cancer research as an integral part of their National Cancer Control Plans.

Our results show that most African countries are heavily (inter)dependent on international collaboration to advance their national cancer research agenda reflecting weak federal national funding for research.^25^ It is already known that many African academic institutions are not financially independent and have instead to look to external research funders to sustain their activities.^26^ The heavy dependence on international collaboration to support African cancer research is also reflected in our results showing that privileged authorship ranking (first or last authorship) is skewed towards non-African authors. Our findings on both collaboration patterns and authorship reflect significant power imbalances. Partly this is driven by the need of Western authors to attain specific positions on academic outputs.^27 28^ In contrast, in many African country’s senior authorship positions (first or last authorship) is contextual and depends on the specific regional academic practices^29 30^ and international collaboration, gives many African researchers greater global visibility and acceptance in high-impact journals compared with domestic-originated research.^31^ Such power imbalances speak to the need for solutions to drive intra-African cancer research collaboration, rather than constantly relying on non-African partnerships.^32^ In addition, journals (national, regional and global) need to develop better policies to ensure power equity in publishing.^33 34^

### Research priorities for African populations

Our findings show that cancer research priorities across Africa broadly mirror those in high-income Western countries. There is a significant under-representation of key cancer domains such as palliative care, implementation science and qualitative research.^19^ The relative decline in childhood cancer research is also a significant issue that, as recent Commissions have pointed out, needs to be addressed.^35^ Childhood cancer research accounts for less than 2% of research analysed in this study yet has the highest potential for improved outcomes and economic impacts on the countries.^36^ In a continent with such a large population who currently present with advanced stages of cancer at diagnosis, there is a clear need to prioritise clinical, palliative and qualitative research to improve outcomes for cancer patients, with involvement of civil society towards community participatory research programmes and research advocacy.

The dominance of breast cancer research across Africa also mirrors global trends with higher funding commitment compared with other cancer types due to high civil society attention, political lobbying and funding.^37^ The under-representation of several other high-burden cancers on the continent, such as liver, cervix and prostate cancers, reflects a narrow research agenda that is not orientated to improving early diagnosis and cost-effective interventions for curable cancers.^38^ For example, sub-Saharan Africa has one of the highest incidences of oesophageal cancer globally but it is woefully under-represented in terms of research, as we have found.^39^

Re-engineering the academic structure of African cancer research institutions would help in establishing a better culture focused on domestic priorities.^40^ This involves a critical look at the funding and compensatory mechanisms for African academic faculty and building a system that supports all aspects of research, including personnel such as research assistants, statisticians and grant managers. Developing a cancer research skills base for example modelled on National Cancer Grid of India’s CREDO programme, which includes a multisectoral stakeholder research taskforce that regularly reviews network cancer research priorities. African adaptations of this model would also help establish a collaborative research mindset which focuses on national needs.^41^ Although similar models exist in a few countries, there is need for pooled regional resources to integrate local efforts. The AORTIC and organisations like the Kenyan Society of Hematology and Oncology (KESHO) could potentially assist in coordinating these priorities. The faculty development program run by AORTIC in collaboration with the Royal College of Physicians and Surgeons of Canada and the Implementation Science and Clinical Trials AORTIC special interest groups are examples of helping to equip African faculty with skills to perform better research. The notion of research as ‘a tedious and painful process’ in Africa is in part due to a lack of adequate training of local ethics committees and institutional review bodies, and acts as a deterrent to many young researchers.^42^

### Gender disparities in cancer research

Gender equality is a core development objective, embedded in the sustainable development goals (goal 5), and essential for equitable research progress. Significant improvements have been made in the closure of gender gaps in sub-Saharan Africa and at 61%, women in sub-Saharan Africa have one of the highest labour force participation rates in the world.^43^

A welcome finding from our research is that African female representation in cancer research is higher than that in most Western countries. However, we found marked country-by-country differences, with some countries such as Mali and Ethiopia having very low levels of female authorship. This suggests a lot more advocacy is needed to create enabling environments to support and promote gender equity in many African countries. This effort can also be amplified by journal support and institutional policies for gender equity in high ranking authorship and leadership positions known to reduce discrepancies.^44^ Our findings echo similar observations on the empowerment of female scientists in Africa.^45^ A greater emphasis on gender inclusivity in African cancer research would also help address some of the major gaps in the treatment of women’s cancers across the continent.^46^

### Concluding comments: Africanising cancer research

Even though the contribution of Africa to global cancer research is increasing much of the funding and research structures reflect a long history of colonialism.^47^ African countries must be encouraged to engage in research that improves cancer services and systems planning. The governance and surveillance of research institutions must point participants to research relevant to regional needs, and reward them appropriately. This is an opportunity for the African Union to implement its commitment to improve cancer research investment in line with Agenda 2063, The Africa We Want. Institutions should be supported to establish regional engagement of cancer researchers with South–South African collaborations. There is a need to redefine measures of research success across African cancer research with outcome indicators that convey the impact on communities, and improvement in capacity and capabilities—building that reflect true national needs.

Non-African, relevant regional scientific evidence should be promoted by journals and sponsors. International collaborators and major sponsors of research should coinvest in cancer research capacity building across Africa perhaps by prioritising ‘neglected countries’ through regional support for less well-established research units and in-service training models to enhance the research capabilities of the existing continental health workforce. In a continent that is struggling with the basic provision of cancer care, and a paucity of adequate health surveillance systems, cancer research outputs should underscore interdisciplinary approaches and be geared towards region-specific research that incorporates and accelerates health systems research and focuses on outcomes and implementation. Existing models of funding impact the choice of research and can be overcome by academic institutions that are independent but obtain national direct government block grants for research.

### Five-point plan for building cancer research across Africa

1. All lower-middle and upper-middle income African countries have committed to spend at least 1% of GDP on gross domestic expenditure in research and development. Of this 1%, 10% should be allocated to cancer research and development across a wider range of disciplines that address national and regional needs.
2. Research needs to be a key component of every national cancer control plan across Africa linked to hypothecated federal funding (see 1).
3. African academic institutions need to develop independent funding sources (federal and philanthropic) that can allow them to drive their own research agenda and become less dependent on international cancer researchers and research funders.
4. New collaborative research models, including capacity building, to enhance international, regional and South–South collaborations need to be developed and implemented, supported by a pan-African cancer research repository.
5. Develop research across Africa including multilingual collaborations that promote research in Francophone and Lusophone countries, ensure ‘orphan’ high burden cancers are represented on the continent and ‘funding deserts’ are minimised

### Reflexivity statement

The research team has been led by SSA authors on behalf of a wide collegium across the continent and including collaborators in high-income countries, particularly the USA and UK. From the outset the research design was codeveloped with African authors leading on setting the parameters of the analysis and the iterative policy discussions with the entire group. African authors are both in first and last position with 14 of the 23 total authorships from African countries. CN from Rwanda is an early career researcher. All the bibliometric data (raw) has been shared with all the coauthors and on publication for every country this data will be made available through multiple websites including AORTIC, KESHO, NCI, USA and KCL GOG. We will also provide infographics for patient and policy advocates for specific countries to help build the case for the inclusion of cancer research into national cancer control policy. The article will also be fully open access as we have funds to support this.

### Patient and public involvement

During the design of the research questions to build the bibliometric analysis, the lead authors utilised their respective patient groups to frame some of the key questions. At subsequent round tables at least 4–6 patient advocates attended, from Kenya, South Africa, Ghana and Rwanda. One patient advocate (BK) put in substantial input to the analysis and drafting and thus is a coauthor. We will provide infographics for patient and policy advocates for specific countries to help build the case for the inclusion of cancer research into national cancer control policy. The article will also be fully open access as we have funds to support this. Dissemination of data will also employ multimedia strategies including social media–twitter spaces blogs, etc to amplify the effects of this data.

**Key messages**

- Qualitative studies dominate the literature on the state of cancer research across continental Africa. This study provides a comprehensive, contemporary quantitative analysis on the state of cancer research and a synthesis of gaps and proposed strategies and directions to proceed with.
- This study provides high-resolution, country-specific data on strengths and weaknesses of cancer research across Africa, by site, by domain as well as sex distribution of authorship. It also provides data with whom and how African authors collaborate internationally. This provides opportunities’ to assess and optimise existing collaborations or perhaps build new ones
- Cancer research intelligence is crucial for patient advocates and policy-makers at a national level to build research into national cancer control planning. It also informs global cancer research funding organisations to support research that is led by and germane to national African priorities.

## Data Availability

Data are available upon reasonable request.
