# Supplementary material for: Cancer research across Africa: a comparative bibliometric analysis
Source: BMJ Glob Health. 2022 Nov 10;7(11):e009849. doi: 10.1136/bmjgh-2022-009849 (PMC9660667; doi:10.1136/bmjgh-2022-009849)
Supplement: Supplementary data [file bmjgh-2022-009849supp001.pdf]

e-Tables

e-Table 1. **African countries, with population (millions) (2019) and GNI/*caput* (2019), USD**. Lan = language(s) spoken or official: A = Anglophone; E = Ethiopian (Amharic); F = Francophone; L = Lusophone (Portuguese); N = North African (Arabic, Berber) **North African countries (bold)** (Source: UN HDI, 2019)

Country	*Lan*	*ISO2*	*Pop*	*GNI/c*	*Country*	*Lan*	*ISO2*	*Pop*	*GNI/c*
Nigeria	A	NG	167	2970	**Tunisia**	**F N**	**TN**	**11.7**	**4035**
Ethiopia	E	ET	86.5	550	Guinea	F	GN	10.5	470
**Egypt**	**N**	**EG**	**84**	**3210**	Somalia	N	SO	9.8	728
Dem Rep Congo	F	CD	69.6	400	Benin	F	BJ	9.4	890
South Africa	A	ZA	50.7	6790	Burundi	F	BI	8.7	270
Tanzania	A	TZ	47.7	920	**Libya**	**N**	**LY**	**6.5**	**7820**
Kenya	A	KE	42.7	1300	Togo	F	TG	6.3	550
Sudan	A N	SD	37.2	1710	Sierra Leone	A	SL	6.1	770
**Algeria**	**F N**	**DZ**	**36.5**	**5490**	Eritrea	A N	ER	5.6	490
Uganda	A	UG	35.6	670	Cent. Afr. Rep.	F	CF	4.6	310
**Morocco**	**F N**	**MA**	**32.6**	**3080**	Liberia	A	LR	4.2	370
Ghana	A	GH	25.5	1590	Rep. of Congo	F	CG	4.2	2720
Mozambique	L	MZ	24.5	620	Mauritania	F	MR	3.6	1370
Madagascar	F	MG	21.9	450	Namibia	A†	NA	2.4	5670
Cote d'Ivoire	F	CI	20.6	1450	Lesotho	A	LS	2.2	1330
Cameroon	A F	CM	20.5	1350	Botswana	A	BW	2.1	7030
Angola	L	AO	20.2	4800	Gambia	A	GM	1.8	460
Burkina Faso	F	BF	17.5	690	Guinea Bissau	L	GW	1.6	590
Niger	F	NE	16.6	420	Gabon	F	GA	1.6	10410
Mali	F	ML	16.3	820	Mauritius	A F	MU	1.3	9720
Malawi	A	MW	15.9	360	Eswatini	A	SZ	1.2	3550
Zambia	A	ZM	13.9	1740	Djibouti	F	DJ	0.9	1030
Senegal	F	SN	13.1	1025	Comoros	F	KM	0.8	1350
Zimbabwe	A	ZW	14.7	840	Equatorial Guinea	F L	GQ	0.7	10210
Chad	F	TD	11.8	980	W. Sahara	N	EH	0.7	1400
Rwanda	A F	RW	11.3	690	Cape Verde	L	CV	0.5	3450

South Sudan	A	SS	10.8	970	São Tomé Principe	L	ST	0.2	1600

e-Table 2. **The disease burden from cancer, percentage of total DALYs, in eight continental regions**. SSA = Sub-Saharan Africa; NA5 = Five North African countries; EEU = Eastern Europe including Russia; LAT = Latin America and Caribbean; WEU = Western Europe; NAM = Canada & USA; OCE = Oceania. Countries ordered by ratio of percentage of all DALYs attributable to cancer in 2019 to that in 2000.

	SSA	NA5	Asia	EEU	LAT	WEU	NAM	OCE	World
**2000 All**	782786	47946	1483654	160745	161348	113057	90297	6326	2798213
**Cancer**	17150	3182	112260	20677	13207	23112	15193	1084	202683
**% total**	2.2	6.6	7.6	12.9	8.2	20.4	16.8	17.1	7.2
**2010 All**	716325	49713	1420242	145137	179252	110079	95952	6806	2673793
**Cancer**	20604	4056	129332	20577	16088	23684	15797	1216	227298
**% total**	2.9	8.2	9.1	14.2	9	21.5	16.5	17.9	8.5
**2019 All**	552997	53667	1373148	124817	177466	119648	118252	11716	2531711
**Cancer**	18758	4867	142722	18951	17201	22446	15523	1506	241974
**% total**	3.4	9.1	10.4	15.2	9.7	18.8	13.1	12.9	9.6
**2019/2000**	1.54	1.37	1.37	1.18	1.18	0.92	0.78	0.75	1.33

e-Table 3. **Codes used to designate research on different anatomical sites and research domains.**

Cancer site	*Code*	*Cancer site*	*Code*	*Research domain*	*Code*
**Bladder**	BLA	**Lung**	LUN	**Systemic therapy**	DRUG
**Blood**	BLO	**Oesophagus**	OES	**Clinical trials**	CLIN
**Breast**	MAM	**Ovary**	OVA	**Diagnosis**	DIAG
**Central nervous system**	CNS	**Pancreas**	PAN	**Epidemiology**	EPID
**Cervix**	CER	**Prostate**	PRO	**Genetics-Biology**	GENE
**Colon & rectum**	COL	**Sarcoma**	SAR	**Paediatric**	PAED
**Eye (inc Rb)**	EYE	**Skin**	SKI	**Palliative care**	PALL
**Head & neck**	MOU	**Stomach**	STO	**Pathology**	PATH
**Kidney**	KID	**Uterus**	UTE	**Biomarker(s)**	PROG
**Liver**	LIV	**Vulva**	VUL	**Quality of life**	QUAL

				**Radiotherapy**	RADI
				**Screening**	SCRE
				**Surgery**	SURG

e-Table 6. **Ratio of observed to expected outputs of cancer research papers on 12 anatomical sites by 13 leading African countries** (2009-20). *For country ISO2 codes, see Table 1; for cancer site trigraphs, see Table 3. Mediterranean countries: black type; SSA countries: grey type*

Ratio	*EG*	*ZA*	*TN*	*MA*	*NG*	*DZ*	*ET*	*KE*	*GH*	*CM*	*UG*	*SD*	*TZ*
MAM	0.92	*1.06*	*0.92*	*0.99*	1.22	**1.33**	1.44	*0.69*	**1.81**	*0.96*	*0.97*	**1.69**	*0.79*
HAE	*1.07*	0.82	**1.17**	**1.21**	*0.81*	*1.11*	**0.16**	*1.27*	*0.85*	*0.80*	*1.12*	*1.28*	*0.91*
LIV	**1.81**	**0.51**	**0.56**	**0.48**	**0.35**	0.43	0.46	**0.13**	*0.56*	*0.80*	0.21	*0.58*	*0.28*
CER	**0.17**	**1.63**	**0.47**	0.74	**1.86**	0.43	**5.78**	**3.78**	**2.63**	1.78	**4.42**	*0.69*	**3.24**
COL	*0.99*	*0.84*	**1.68**	*1.11*	0.70	*1.12*	*0.53*	0.23	*0.57*	0.30	0.21	1.81	*1.36*
LEU	**1.39**	**0.49**	*1.13*	*0.94*	0.68	*1.38*	0.29	0.31	*0.59*	*0.56*	0.05	1.91	*0.53*
MYE	**1.27**	0.79	*0.81*	0.69	*0.90*	*0.88*	**0.06**	*0.91*	*0.41*	*0.46*	*1.08*	*0.89*	*1.26*
CNS	*0.91*	0.71	**1.57**	**1.86**	*1.02*	**1.77**	*0.49*	*0.49*	*0.85*	*0.50*	*0.25*	*0.78*	*0.18*
HEN	0.88	*0.99*	*1.16*	**1.71**	**1.59**	*0.57*	*0.62*	*0.41*	*0.93*	0.04	*0.33*	*1.30*	*0.79*
LUN	*0.98*	*1.01*	**1.72**	**2.08**	**0.34**	*1.00*	*0.48*	0.09	0.15	0.18	0.06	*0.18*	*0.08*
PRO	**0.67**	*1.14*	0.70	**0.46**	**2.17**	*1.14*	*0.63*	*0.70*	**2.17**	*1.20*	*0.96*	*1.72*	*0.79*
LYM	**0.76**	*1.11*	*1.21*	**1.70**	0.56	*0.95*	0.01	**2.68**	*1.60*	*1.36*	**2.55**	*0.78*	*0.82*

Cells where p < 0.5% in **bold** type; where p < 5% in roman type; where not significant (p > 5%) in *small italics.*

e-Table 7. **Ratio of observed to expected outputs of cancer research papers in 13 domains by 13 African countries with the largest cancer research outputs** (2009-20. *For country ISO2 codes, see Table 1; for domain tetragraphs, see Table 3. North African countries: black type; SSA countries: grey type*

Ratio	*EG*	*ZA*	*TN*	*MA*	*NG*	*DZ*	*ET*	*KE*	*GH*	*CM*	*UG*	*SD*	*TZ*
GENE	**1.18**	**0.72**	**1.52**	*0.87*	**0.43**	1.34	**0.50**	0.59	*0.85*	0.58	**0.42**	**1.58**	0.51
DRUG	**1.22**	*0.92*	**0.53**	**0.47**	**0.66**	*0.81*	*0.76*	*0.77*	**0.38**	*0.60*	0.49	*0.88*	0.37
PROG	**1.28**	**0.75**	*0.89*	**0.49**	**0.62**	0.56	0.50	*0.67*	*0.88*	*0.75*	*0.90*	*0.76*	*0.76*
SURG	**1.15**	1.14	*0.91*	**1.65**	*0.99*	**0.42**	**0.32**	*1.38*	*0.79*	*1.27*	*0.58*	*1.15*	**1.89**
PATH	**1.18**	**0.73**	**1.45**	*0.89*	*0.81*	**1.68**	**0.17**	*0.71*	*1.01*	*0.64*	*0.45*	*1.26*	*0.79*
DIAG	**1.28**	**0.73**	*0.86*	*1.04*	*0.92*	*0.94*	*1.21*	*1.32*	*1.29*	*0.54*	*1.23*	*0.85*	*1.14*

EPID	**0.59**	*0.91*	**1.39**	*1.07*	**1.49**	**1.85**	*1.28*	*1.50*	*1.51*	*1.23*	**2.47**	*0.86*	**2.46**
RADI	**0.78**	*0.84*	*1.25*	**1.56**	*0.76*	*1.21*	0.33	0.08	1.86	*0.54*	*0.32*	*0.71*	*0.89*
SCRE	**0.49**	*1.06*	**0.48**	0.66	**2.39**	*1.58*	**5.09**	**2.77**	**3.11**	**2.18**	**3.69**	*1.12*	**3.48**
CLIN	1.20	**0.57**	**0.31**	**0.11**	**0.34**	*0.22*	*0.77*	*1.37*	*0.71*	*0.39*	*0.74*	*0.14*	*0.73*
PALL	*1.05*	*1.28*	0.41	*0.63*	**2.01**	*0.36*	**5.09**	**2.78**	2.23	*1.62*	*1.52*	*0.56*	*0.00*
QUAL	**0.54**	*0.90*	0.33	*0.82*	**3.14**	*0.53*	**8.89**	*1.41*	2.70	*0.33*	*2.07*	*0.00*	*0.47*
TOBA	0.53	*1.03*	**3.88**	*0.41*	*0.64*	*1.72*	*0.32*	*0.00*	*0.00*	*1.81*	*2.72*	*0.00*	*0.00*

Cells where p < 0.5% in **bold** type; where p < 5% in roman type; where not significant (p > 5%) in *small italics*

e-Table 9. **The sex determination of cancer researchers in first position** (numbers of individual contributions), 2009-20, for countries with at least 15 such papers. F = females, M = males, U = unknown, F/(M+F) = percent of sexed names that are female. Data rows tinted are for North African countries.

First A	*F*	*M*	*U*	*F/(M+F)*	*Total*	*First A*	*F*	*M*	*U*	*F/(M+F)*	*Total*
**TN**	933	495	175	65.3	1603	MW	21	38	10	35.6	69
ZM	21	12	10	63.6	43	GH	49	103	17	32.2	169
GA	9	6	3	60.0	18	KE	58	122	14	32.2	194
**DZ**	158	109	46	59.2	313	UG	40	87	11	31.5	138
MZ	9	9	3	50.0	21	ZW	11	26	10	29.7	47
ZA	789	858	393	47.9	2040	**LY**	12	29	12	29.3	53
MU	9	10	6	47.4	25	SD	31	80	12	27.9	123
**EG**	3026	3423	303	46.9	6752	SN	19	64	26	22.9	109
**Total**	**5985**	**7202**	**1573**	**45.4**	**14760**	NG	175	606	148	22.4	929
MG	4	5	9	44.4	18	CM	30	111	19	21.3	160
**MA**	469	617	218	43.2	1304	TZ	18	69	10	20.7	97
BW	12	16	5	42.9	33	ML	4	23	4	14.8	31
BF	13	22	13	37.1	48	ET	24	161	42	13.0	227
RW	13	22		37.1	35	TG	1	12	20	7.7	33
CI	14	25	20	35.9	59						

e-Table 10. **The sex determination of cancer researchers** in last position (numbers of individual contributions), 2009-20, for countries with at least 14 such papers. F = females, M = males, U = unknown, F/(M+F) = percent of sexed names that are female. Data rows tinted are for North African countries.

Last A	*F*	*M*	*U*	*F/(M+F)*	*Total*	*Last A*	*F*	*M*	*U*	*F/(M+F)*	*Total*

MG	4	3	9	57.1	16	RW	6	15		28.6	21
MZ	6	7	1	46.2	14	**MA**	298	802	143	27.1	1243
MU	11	13	3	45.8	27	TZ	15	56	8	21.1	79
BW	14	18	7	43.8	39	NG	131	500	170	20.8	801
ZA	728	968	312	42.9	2008	SN	15	64	29	19.0	108
**LY**	16	22	10	42.1	48	ZM	7	32	7	17.9	46
**EG**	2536	3539	336	41.7	6411	CM	16	75	14	17.6	105
TG	10	14	9	41.7	33	BF	4	20	5	16.7	29
ZW	12	18	10	40.0	40	SD	14	72	7	16.3	93
MW	24	42	6	36.4	72	GH	19	116	11	14.1	146
**Total**	**4455**	**7886**	**1343**	**36.1**	**13684**	GA	1	8	6	11.1	15
**DZ**	63	117	34	35.0	214	ML	2	18	2	10.0	22
KE	42	96	8	30.4	146	ET	13	157	37	7.6	207
**TN**	411	952	126	30.2	1489	CI	2	31	15	6.1	48
UG	30	70	10	30.0	110						

e-Table 11. **The sex determination of sole cancer researchers** (numbers of individual contributions), 2009-20, for countries with at least 5 such papers. F = females, M = males, U = unknown, F/(M+F) = percent of sexed names that are female. Data rows tinted are for North African countries.

Sole A	*F*	*M*	*U*	*F/(M+F)*	*Total*		*Sole A*	*F*	*M*	*U*	*F/(M+F)*	*Total*
**TN**	4	2		66.7	6		KE	1	4		20.0	5
**MA**	3	2	2	60.0	7		GH	1	6		14.3	7
ZA	40	43	14	48.2	97		NG	3	18	5	14.3	26
**Total**	**127**	**322**	**42**	**28.3**	**491**		CM		8		0.0	8
**EG**	72	214	13	25.2	299		ET		7	1	0.0	8
**DZ**	1	4	3	20.0	8		SD		5		0.0	5

e-Table 12a **Acknowledgement of research funding organisations by linguistic region** (absolute number)

Funders for five North African countries	***N***
US National Institutes of Health (NIH) other	836
US National Cancer Institute (NCI)	811
Egyptian Government	600
King Saud University (Saudi Arabia)	425
Tunisian government	401
Japanese government	400
King Abdulaziz City and University (Saudi Arabia)	189
European Union (EU)	167
Cairo University	121
Cancer Research UK	85
Egyptian Academy of Sciences (Science & Technology Dev't Fund)	84
National Natural Science Foundation of China (NSFC)	84
UK National Institute for Health Research (NIHR)	83
German Research Foundation (DFG)	78
US National Science Foundation (NSF)	75
German Academic Exchange Service (DAAD)	71
UK Medical Research Council	71
Alexander von Humboldt foundation	64
Algerian government	62
Elekta AB	61
Institut National de la Sante et de la Recherche Medicale (INSERM)	61
King Khalid University (Saudi Arabia)	54
Korea Institute of Science and Technology (KIST)	51
Moroccan government	45
National Research Centre, Cairo	36
Wellcome Trust	36
Centre National de la Recherche Scientifique (CNRS)	30
Libyan government	26

e-Table-12b

Funders for Francophone SSA countries	***N***
US National Institutes of Health (NIH) other	263
US National Cancer Institute (NCI)	85
US NIH Fogarty International Center (FIC)	64
Alexander von Humboldt Foundation (Germany)	52
US National Science Foundation (NSF)	43
European Union (EU)	30
UK Medical Research Council (MRC)	27
Wellcome Trust	25
National Natural Science Foundation of China (NSFC)	22
US Department of Agriculture (USDA)	19
Bill & Melinda Gates Foundation	18
German Research Foundation (DFG)	18

e-Table12c

Funders for Anglophone SSA countries	***N***
US National Institutes of Health (NIH) other	1072
US National Cancer Institute (NCI)	811
US NIH Fogarty International Center (FIC)	333
National Natural Science Foundation of China (NSFC) incl. provinces	110
Wellcome Trust	107
UK Medical Research Council	79
Susan G. Komen Breast Cancer Foundation	65
Alexander von Humboldt Foundation (Germany)	63
Bill & Melinda Gates Foundation	60
European Union (EU)	56
Japanese Ministry of Education, Culture, Sports, Science and Technology	54
Cancer Research UK	49
American Cancer Society	34
Breast Cancer Research Foundation (US)	30

e-Table 12d

Funders for Lusophone SSA countries	***N***
Portuguese Foundation for Science and Technology (FCT)	20
US National Institutes of Health (NIH) other	19
US NIH Fogarty International Center (FIC)	12
European Union (EU)	7
Fundacao Calouste Gulbenkian	6
US NIH National Cancer Institute (NCI)	3
Wellcome Trust	3

e-Table12d

Funders for South Africa	***N***
South African National Research Foundation (NRF)	910
US National Institutes of Health (NIH) other	539
South African universities	518
US National Cancer Institute (NCI)	451
Cancer Association of South Africa (CANSA)	386
South African Medical Research Council (SAMRC)	338
European Union (EU)	171
Cancer Research UK	132
UK Medical Research Council	114
South African Department of Science and Technology (DST)	98
UK National Institute for Health Research (NIHR)	87
National Health and Medical Research Council of Australia	81
Department of Science and Technology, Government of India	58
