# Supplementary figures and images for: Cancer research across Africa: a comparative bibliometric analysis
Source: BMJ Glob Health. 2022 Nov 10;7(11):e009849. doi: 10.1136/bmjgh-2022-009849 (PMC9660667; doi:10.1136/bmjgh-2022-009849)

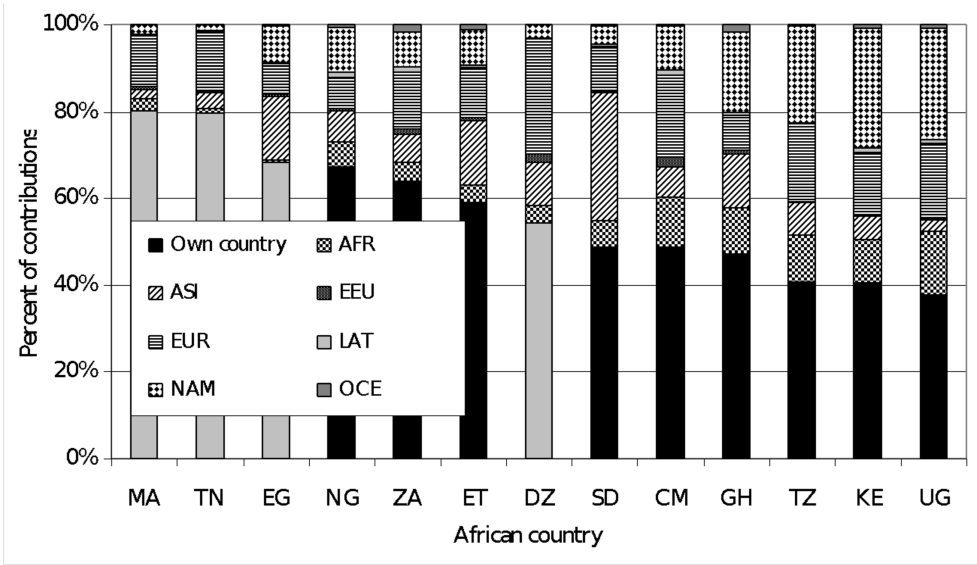

Supplement: Supplementary data [file bmjgh-2022-009849supp002.pdf]

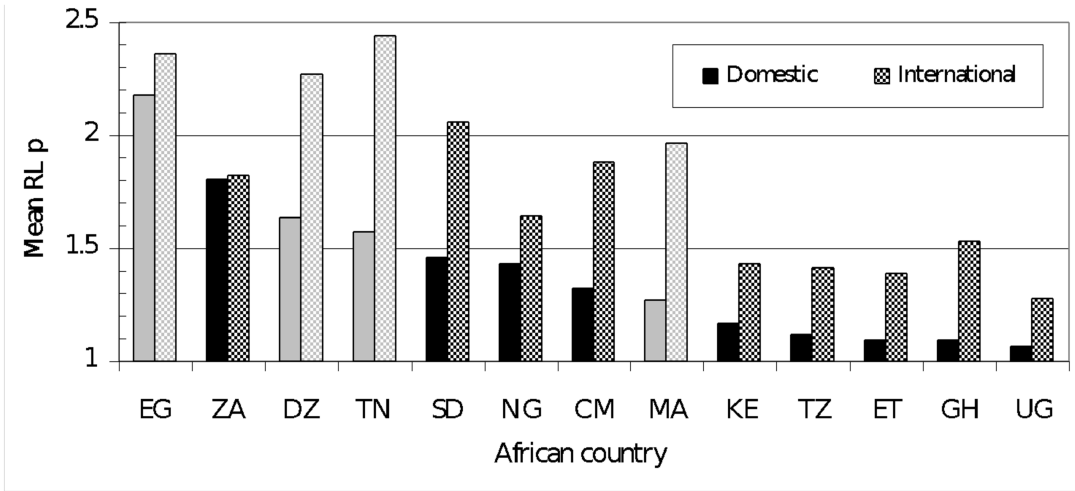

Supplement: Supplementary data [file bmjgh-2022-009849supp003.pdf]
